# Maternal fat-soluble vitamin trajectories and infant birth weight in individuals with overweight or obesity

**DOI:** 10.3389/fendo.2026.1809102

**Published:** 2026-04-15

**Authors:** Astrid Kamilla Stunes, Unni Syversen, Anna Hundere Øvreseth, Ingvild Tapio Kinge, Siv Mørkved, Kjell Åsmund Salvesen, Kirsti Krohn Garnæs, Trine Moholdt

**Affiliations:** 1 Department of Clinical and Molecular Medicine, Faculty of Medicine and Health Sciences, Norwegian University of Science and Technology (NTNU), Trondheim, Norway; 2 Center for Oral Health Services and Research, Mid-Norway (TkMidt), Trondheim, Norway; 3 Department of Public Health and Nursing, Faculty of Medicine and Health Sciences, Norwegian University of Science and Technology (NTNU), Trondheim, Norway; 4 Department of Obstetrics and Gynecology, St Olav’s Hospital, Trondheim University Hospital, Trondheim, Norway; 5 Department of Circulation and Medical Imaging, Faculty of Medicine and Health Sciences, Norwegian University of Science and Technology (NTNU), Trondheim, Norway

**Keywords:** birth weight, fat-soluble vitamins, obesity, overweight, pregnancy, vitamin A, vitamin D, vitamin E

## Abstract

**Background:**

Birth weight is a strong determinant of long-term metabolic health, with both low birth weight and macrosomia linked to increased cardiometabolic risk. Fat-soluble vitamins A, D, and E modulate pathways relevant to fetal growth, however, their trajectories and potential associations with birth weight in pregnant individuals with overweight or obesity remain scarcely characterized.

**Objective:**

To examine maternal vitamins A (retinol), D (25(OH)D), and E (α-tocopherol) concentrations during the second and third trimesters of pregnancy and their associations with birth weight, in individuals with pre-pregnancy overweight or obesity.

**Methods:**

This secondary analysis of the Exercise Training in Pregnancy Trial (ETIP) study included 57 mother-infant pairs with available vitamin measurements in the second and third trimesters. Plasma retinol and α-tocopherol were measured by high performance liquid chromatography, and serum 25(OH)D by liquid chromatography-tandem mass spectrometry. Birth weight was classified as normal (2500 – < 4000 g) or macrosomic (≥ 4000 g). Circulating vitamin concentrations between trimesters were compared using the Wilcoxon signed-rank test and associations between vitamins and birth weight were examined using multivariate linear regression models.

**Results:**

From the second to the third trimester, mean maternal retinol and 25(OH)D concentrations declined significantly (retinol: 1.52 (SD = 0.37) to 1.32 (SD = 0.33) µmol/L; 25(OH)D: 73.7 (SD = 30.0) to 63.3 (SD = 25.0) nmol/L), whereas α-tocopherol increased from 34.6 (SD = 7.1) to 46.3 (SD = 10.0) µmol/L. In the third trimester, 19.3% had vitamin A insufficiency, while vitamin D deficiency and insufficiency affected 31.6% and 33.3%, respectively. Macrosomia occurred in 43.9% of infants and 56.1% had birth weight within the normal range. Maternal vitamins A, D, and E were not associated with birth weight, and no vitamin A and D interaction was observed.

**Conclusion:**

In pregnant individuals with overweight or obesity, maternal vitamin A and D concentrations declined across pregnancy, while vitamin E increased. Vitamin A insufficiency, vitamin D deficiency/insufficiency, and macrosomia were common. Maternal fat-soluble vitamin levels were not independently associated with birth weight, suggesting that vitamin status during mid- to late pregnancy may not be a major determinant of fetal growth in this metabolically high-risk population.

## Introduction

Birth weight is a key determinant of long-term cardiometabolic health. Infants born small or large for gestational age or with macrosomia have an increased risk of developing obesity, type 2 diabetes, and cardiovascular disease later in life (1). Macrosomia is strongly associated with childhood and adolescent obesity, suggesting that early-life growth trajectories play an important role in lifelong metabolic risk (2). Fetal growth is influenced by a complex interplay between maternal nutritional status, endocrine regulation, and placental function. Among nutritional factors, fat-soluble vitamins can bey relevant due to their roles in cellular differentiation, hormone signaling, and oxidative balance during pregnancy (3). Current data do not definitively establish how maternal concentrations of vitamins A, D, and E affect birth weight, particularly in the context of maternal overweight or obesity. Adequate levels of these vitamins may support normal fetal growth, whereas both deficiency and excess have been linked to adverse pregnancy outcomes, including low birth weight and macrosomia (4–6).

Vitamin A is essential for fetal organ development and growth (3, 7). Elevated maternal vitamin A has been found to be associated with an increased risk of low birth weight, whereas low maternal concentrations are associated with high birth weight and macrosomia (6, 8, 9). Vitamin D acts as a pleiotropic hormone involved in skeletal development and metabolic regulation, and maternal deficiency has been linked to both low birth weight and, in some populations, an increased risk of macrosomia (10–12). Vitamin E acts as a lipid-soluble antioxidant that supports placental function, and higher maternal α-tocopherol levels have been linked to greater fetal growth through effects on angiogenesis and nutrient transfer (13). Evidence on vitamin E and birth weight is limited, but large cohort studies report that higher maternal concentrations are associated with increased birth weight and a greater risk of macrosomia (6), alongside a decreased risk of low birth weight (13).

Maternal overweight and obesity alter nutrient handling during pregnancy, including the metabolism and bioavailability of fat-soluble vitamins (14–16). Obesity is consistently associated with vitamin D deficiency due to sequestration in adipose tissue (17), whereas circulating vitamin A concentrations remain relatively stable because of tight hepatic regulation (18), and vitamin E concentrations may be elevated due to increased lipid transport (19). Together, these obesity-related alterations in fat-soluble vitamin metabolism highlight a critical knowledge gap regarding their role in regulating fetal growth in metabolically high-risk pregnancies.

To address these gaps, this study aimed to examine vitamin A, D and E status during the second and third trimesters of pregnancy and whether these fat-soluble vitamins were associated with birth weight in individuals with pre-pregnancy overweight or obesity living in a high-income setting. Specifically, we investigated whether maternal concentrations of these vitamins were associated with fetal growth and thereby increasing the risk of macrosomia. By focusing on a metabolically high-risk population, this study seeks to contribute to early-life programming research and improve understanding of how maternal vitamin status might influence long-term disease risk through its effects on birth weight.

## Materials and methods

### Study design, setting, and participants

This study used data from participants enrolled in the randomized controlled trial (RCT) Exercise Training in Pregnancy (ETIP), conducted in Norway between 2010 and 2015 (20). The primary aim of the original RCT was to evaluate the effect of exercise training on gestational weight gain in individuals with pre-pregnancy obesity, with intention-to-treat analyses and supplementary per protocol analyses (20). Participants who were included in gestational week 12–18 were randomly assigned to either an exercise intervention or standard antenatal care (20, 21). Eligibility criteria were individuals aged ≥ 18 years with a pre-pregnancy body mass index (BMI) ≥ 28 kg/m^2^ who were carrying a singleton fetus and were between gestational weeks 12 and 18 at inclusion. Exclusion criteria were medical conditions that could affect participation, high risk of preterm delivery, and engaging in regular exercise training before inclusion. Participants were recruited via invitations distributed alongside routine hospital ultrasound appointment notices, as well as through targeted online advertisements. Individuals who expressed interest in participating contacted the study investigators and were subsequently screened for eligibility via telephone. The study provided participants with infant food worth US $65, and all women gave written informed consent for publication of their case details. The exercise group received standard maternity care plus a structured, supervised training program from early pregnancy to delivery, including weekly hospital sessions, home exercises, pelvic-floor training, weight-gain guidance, and one motivational interview, while adherence was monitored throughout. The control group received ordinary Norwegian maternity care and continued their usual daily activities without restrictions on independent exercise. More details are described in the original study protocol and study (20, 21).

**Figure 1:**
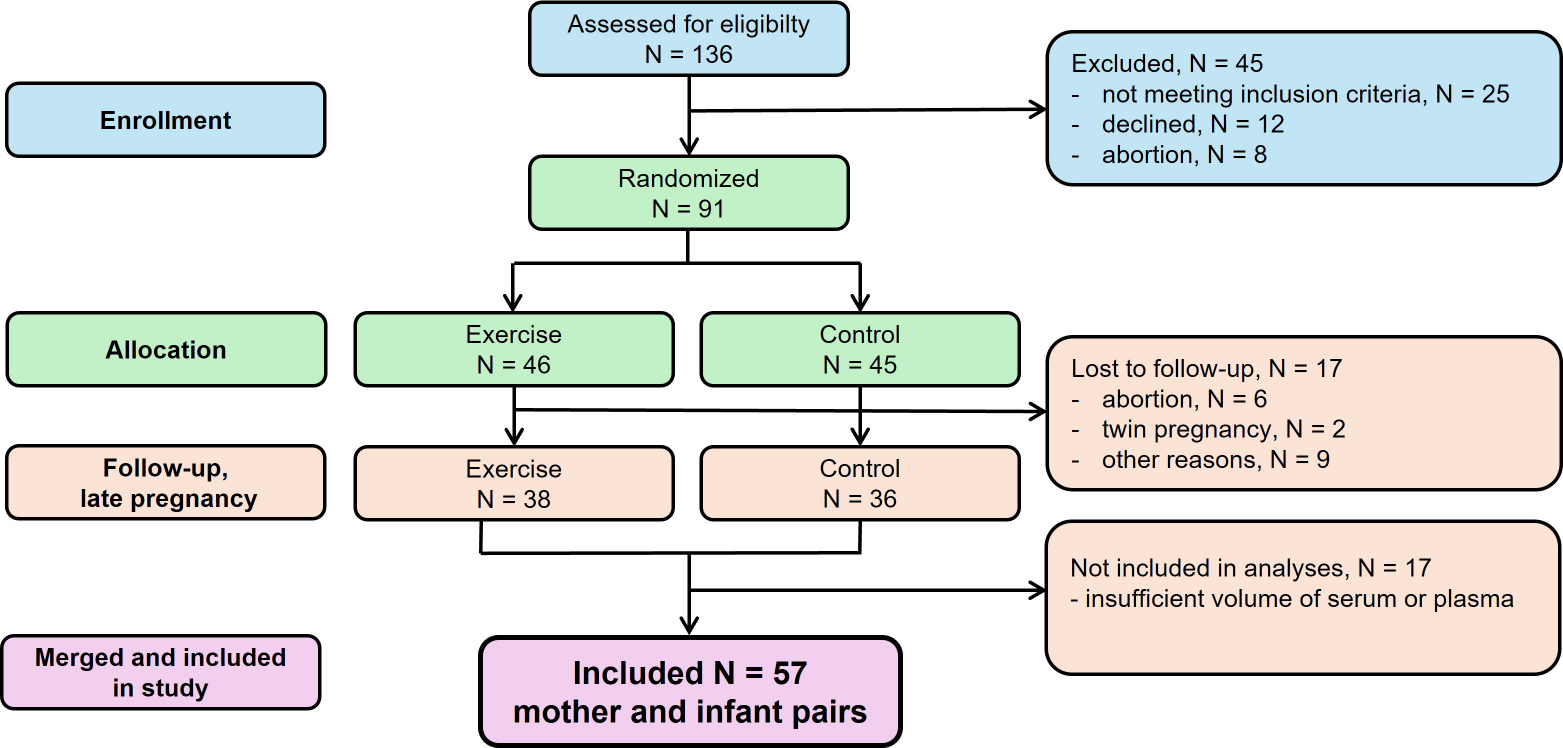
Flow chart of the study.

In this analysis, we included 57 participants from the ETIP study with available plasma and serum samples from the second and third trimesters for analysis of retinol, 25(OH)D and α-tocopherol, and infant birth weight. A flow chart of the study is shown in **Figure 1**. We combined participants from both original RCT groups for the present analysis, as they were comparable with respect to the exposures (maternal vitamin concentrations in the second and third trimesters) and the main outcome (birth weight).

### Data collection

All participants underwent the same testing protocol at inclusion in the second trimester (gestational week 12 – 18) and the third trimester (gestational week 34 – 37). We measured height at inclusion with a wall-mounted Seca 222 stadiometer and maternal body weight in the second and third trimesters using a calibrated electronic scale (Seca 770, Medema, Norway). At inclusion, participants self-reported pre-pregnancy body weight, parity, highest completed education level, and smoking status, and we calculated pre-pregnancy BMI (kg/m^2^).

We collected venous blood samples after a minimum 10-hour overnight fast using standard venipuncture procedures. Fasting blood glucose was measured, and serum and plasma were subsequently aliquoted and stored at -80 °C until analysis. Participants subsequently completed a 75-g oral glucose tolerance test (OGTT), and we measured plasma glucose concentrations again after 2 hours. We measured systolic and diastolic blood pressure on the right arm after 15 minutes of supine rest using a CASMED 740 MAXNIBP (CAS Medical Systems). The average of three measurements taken at 2-minute intervals was used for the analysis. Maternity ward staff documented gestational age, maternal pre-delivery body weight, and newborn birth weight, which we later retrieved from medical records.

### Plasma and serum analyses

Plasma retinol (all-trans retinol) and α-tocopherol were analyzed in all samples simultaneously using high performance liquid chromatography (HPLC) at the Department of Medical Biochemistry, Faculty of Medicine, University of Oslo. Serum 25(OH)D was analyzed simultaneously in all samples using liquid chromatography-tandem mass spectrometry (LC-MS/MS) at the Hormone Laboratory, Oslo University Hospital, Aker Hospital.

### Classifications

We defined gestational diabetes mellitus (GDM) according to the 2006 World Health Organization (WHO) criteria as fasting plasma glucose ≥ 7.0 mmol/L and/or 2-hour plasma glucose ≥ 11.1 mmol/L following OGTT in individuals without pregestational diabetes (22). Gestational hypertension (HTN) was defined as systolic blood pressure > 140 mm Hg and/or diastolic blood pressure > 90 mm Hg in individuals without pregestational HTN (23). We classified pre-pregnancy BMI according to WHO categories as overweight (≥ 25.0 – < 30.0 kg/m^2^), obesity class I (≥ 30.0 – < 35.0 kg/m^2^), and obesity class II-III (≥ 35.0 kg/m^2^) (24). The categorization of gestational weight gain was according to the Institute of Medicine (IOM) guidelines (**Supplementary Table 1**) (25).

For vitamin A, we classified plasma retinol concentrations according to established cut-offs: ≥ 2.45 µmol/L as excessive, ≥ 1.05 – < 2.45 µmol/L as sufficient, ≥ 0.70 – < 1.05 μmol/L as insufficient, and > 0.35 – < 0.70 μmol/L as deficient (26). This classification is also applicable in pregnancy (27). Because maternal plasma retinol values were reported with one decimal, we applied cut-offs accordingly. We classified serum 25(OH)D concentrations in line with the Endocrine Society 2011 guidelines: ≥ 175 nmol/L as excessive, ≥ 75 nmol/L as sufficient, ≥ 50 – < 75 nmol/L as insufficient, and < 50 nmol/L as deficient (28). Sample seasons for vitamin D were defined as Summer from April to September and Winter from October to March. As no consensus exists for optimal vitamin E concentrations during pregnancy, we used previously reported cut-offs for plasma α-tocopherol: ≥ 46 µmol/L as excessive, ≥ 12 – < 46 µmol/L as sufficient, and < 12 µmol/L as deficient (6, 29, 30). We categorized birth weight as within normal weight range if it was ≥ 2500 – < 4000 g and as macrosomia if it was ≥ 4000 g (31, 32).

### Statistical analysis

We assessed data normality and homogeneity of variance using the Shapiro-Wilk test and Levene’s test, respectively. Differences in the circulating concentrations of vitamins A, D and E between the second and third trimesters were compared using the Wilcoxon signed-rank test.

Associations between maternal vitamin A, D, and E, as the average concentration of both trimesters and as second and third trimesters concentrations, and birth weight were assessed using linear regression models. We assessed assumptions for linear regression by examining linearity, normality of residuals, homoscedasticity, independence of errors, and multicollinearity. Diagnostic plots were visually inspected, and multicollinearity was evaluated using variance inflation factors. No substantial violations were identified. We selected covariates using a predefined directed acyclic graph (DAG), which we developed from prior empirical evidence and biological plausibility to identify a minimally sufficient adjustment set for the associations between maternal vitamin concentrations and infant birth weight (**Supplementary Figure 1**). Model 1 adjusted for the potential confounders and co-variates: maternal age, pre-pregnancy BMI, parity (≥ 1 vs. 0), education (no university vs. university), original RCT group allocation, and smoking status. We imputed missing pre-pregnancy BMI values (n = 2) with the cohort median and classified missing smoking data (n = 1) as non-smoking. Model 2 further adjusted for potential mediators, including gestational weight gain category (above vs. recommended/below), gestational length (weeks), GDM (yes in the second and/or third trimester vs. neither), and gestational HTN (yes in the second and/or third trimester vs. neither). To explore a potential synergistic effect between the vitamins A and D on birth weight, we constructed an additional regression model using the product of centered (z-scores) of mean vitamin A and D concentrations from both trimesters. Results are presented as crude and adjusted β coefficients with 95% confidence intervals. We performed all statistical analyses using SPSS Statistics version 30.0 (IBM Corp., NT, USA) and GraphPad Prism version 10.6.1 (GraphPad Software, MA, USA). Statistical significance was defined as p < 0.05.

## Results

### Participants

A total of 91 participants were enrolled in the original RCT. Complete data on maternal vitamin concentrations in the second and third trimesters and infant birth weight were available for 57 mother-infant pairs, who were included in this study (**Figure 1**). **Table 1** summarizes baseline and pregnancy characteristics of the study population. Participants were enrolled in the second trimester at a median gestational age of 15 weeks, and the mean maternal age was 31.4 (SD = 3.5) years. Nearly half of the participants (47.3%) were nulliparous, 38 (66.7%) had education at university level, and eight (14.3%) participants reported smoking during pregnancy. At inclusion, the mean BMI was 34.8 (SD = 4.5) kg/m^2^.

**Table 1:** Characteristics of the study population.

N = 57, Characteristics	Mean ± SD	Median (IQR)	Number [%]
Pre-pregnancy			
Weight, kg^a^	96.1 ± 14.2	94.0 (21)	
Height, cm	168.1 ± 5.5	168.0 (8.0)	
BMI, kg/m^2,a^	34.00 ± 4.60	33.25 (6.05)	
Parity			
0			27 [47.3]
1			25 [43.9]
2			5 [8.8]
Education			
High school			15 [26.3]
University ≥ 4 years			13 [22.8]
University > 4 years			25 [43.9]
Other			4 [7.0]
Inclusion, second trimester			
Gestational week	14.7 ± 2.8	15 (4)	
Age, years	31.4 ± 3.5	32.0 (5.0)	
Weight, kg	98.4 ± 13.5	94.2 (17.8)	
BMI, kg/m^2^	34.8 ± 4.5	33.4 (6.4)	
Smoking**^a^**			8 [14.3]
GDM*			4 [7.0]
G-HTN**,**^a^**			6 [10.7]
Original RCT group (20, 21)			
Exercise			31 [54.4]
Control			26 [45.6]
Third trimester			
Gestational week	35.5 ± 1.1	35 (2)	
Weight, kg	105.89 ± 17.8	104.6 (19.3)	
BMI, kg/m^2^	37. 5 ± 4.6	36.4 (5.6)	
GDM*,**^a^**			10 [18.2]
G-HTN**,**^b^**			8 [14.5]
Delivery			
Gestational week	39.7 ± 1.2	40 (2)	
Weight, kg	108.3 ± 14.7	107.6 (19.0)	
Infant birth weight, g	3877.6 ± 434.1	3880 (685)	
Normal weight range			32 [56.1]
Macrosomia			25 [43.9]
Infant sex^c^			
Female			26 [56.5]
Male			20 [43.5]
Total gestational weight gain, kg***^,a^	11.77 ± 9.80		
Weekly gestational weight gain, kg^#^	0.40 ± 0.22		
Gestational weight gain category^##^			
Under			6 [10.5]
Within			8 [14.0]
Over			43 [75.5]

Normal weight range: ≥ 2500 – < 4000 g, Macrosomia: ≥ 4000 g.

**^a^**missing data N = 2, **^b^**missing data N = 1, ^c^missing data N = 11, BMI, body mass index.

*GDM, Gestational Diabetes Mellitus, fasting plasma glucose ≥ 7.0 mmol/L, and/or 2 hours glucose ≥ 11.1 mmol/L after an Oral Glucose Tolerance Test

**G-HTN, Gestational Hypertension, systolic blood pressure ≥ 140 mmHg and/or diastolic blood pressure ≥ 90 mmHg

***Gestational weight gain from pre-pregnancy to pre-delivery

**^#^**Gestational weight gain per week from second trimester to pre-delivery

**^##^**Gestational weight gain category based on pre-pregnancy body mass index (BMI) as recommended by Institute of Medicine (IOM) guidelines (25).

At follow-up in the third trimester (median gestational week 35), mean BMI had increased to 37.5 (SD = 4.6) kg/m^2^. In the second trimester, four participants (7.0%) were diagnosed with GDM and six (10.7%) with gestational HTN, these numbers increased to 10 (18.2%) and eight (14.5%), respectively, in the third trimester. None of the participants were diagnosed with preeclampsia. From the second trimester until delivery, mean gestational weight gain was 0.40 (SD = 0.22) kg/week. According to the IOM guidelines for gestational weight gain (**Supplementary Table 1**) (25), 43 participants (75.5%) exceeded the recommended weight gain, eight (14%) gained within the recommended range, and six (10.5%) gained less than recommended. The median gestational length was 40 weeks (range 36 – 42).

One infant was born at 36 weeks; all others were born at term. The mean birth weight was 3878 (SD = 434.1) g, and no infants were classified as having low birth weight (< 2500 g). Thirty-two infants (56.2%) had birth weight within the normal range, whereas 25 infants (43.9%) had a birth weight ≥ 4000 g and were classified as macrosomic. The distribution in infant sex was 43.5% female and 56.5% male.

### Maternal vitamin concentrations

#### Vitamin A (plasma retinol)

Mean plasma retinol concentration was 1.52 (SD = 0.37) µmol/L in the second trimester and declined significantly to 1.32 (SD = 0.33) µmol/L in the third trimester (**Table 2**). One participant had plasma retinol concentrations exceeding the upper reference limit (≥ 2.5 µmol/L) during the second trimester. No cases of vitamin A deficiency were observed in either trimester. Vitamin A insufficiency was present in 14% of the participants in the second trimester and increased to 19.3% in the third trimester.

**Table 2:** Maternal levels of vitamin A, D and E in the second and third trimesters of pregnancy.

N = 57	Second trimester	Third trimester	Median differences between trimesters	P-value
Vitamin A, p-retinol, µmol/L	1.52 ± 0.37 1.5 (0.4)	1.32 ± 0.33 1.3 (0.4)	-0.2	< 0.001
< 0.7, deficiency	–	–		
≥ 0.7 – < 1.1, insufficiency	8 [14.0]	11 [19.3]		
≥ 1.1 – 2.5, sufficiency	48 [84.2]	46 [80.7]		
≥ 2.5, excessive	1 [1.8]	–		
Vitamin D, s-25(OH)D, nmol/L	73.67 ± 30.00 74.7 (51.7)	63.64 ± 24.96 57.4 (35.1)	-10.1	0.018
< 50, deficiency	16 [28.1]	18 [31.6]		
≥ 50 – < 75 insufficiency	13 [22.8]	19 [33.3]		
≥ 75 – < 175, sufficiency	28 [49.1]	20 [35.1]		
≥ 175, excessive	–	–		
Vitamin E, p-α-tocopherol, µmol/L	34.61 ± 7.14 35.0 (9.0)	46.28 ± 10.02 46.0 (12.0)	11.0	< 0.001
< 12, deficiency	–	–		
≥ 12 – < 46, sufficiency	54 [94.7]	28 [49.1]		
≥ 46, excessive	3 [5.3]	29 [50.9]		

Data are in mean ± standard deviation, median (interquartile range) or number [percentage].

p, plasma; s, serum.

#### Vitamin D (serum 25(OH)D)

Mean serum 25(OH)D concentration was 73.7 nmol/L (SD = 30.0) nmol/L in the second trimester and decreased significantly to 63.3 (SD = 25.0) nmol/L in the third trimester (**Table 2**). No participants had excessive serum 25(OH)D concentrations in either trimester. In the second trimester, 28.1% of participants were classified as vitamin D deficient and 22.8% as vitamin D insufficient. These proportions increased in the third trimester to 31.6% and 33.3%, respectively. No seasonal differences in serum 25(OH)D concentrations were observed in any trimester.

#### Vitamin E (plasma α-tocopherol)

Mean plasma α-tocopherol concentration increased significantly from 34.6 (SD = 7.1) µmol/L in the second trimester to 46.3 (SD = 10.0) µmol/L by the third trimester (**Table 2**). No participants were classified as vitamin E deficient. However, three participants (5.3%) had excessive vitamin E concentrations in the second trimester, increasing markedly to 29 (50.9%) by the third trimester.

### Associations between maternal vitamin concentrations and birth weight

Selected maternal and pregnancy characteristics stratified by birth weight category, are shown in **Supplementary Table 2**. Regression analyses assessing associations between maternal vitamin concentrations and birth weight are presented in **Tables 3**, **4**; **Supplementary Table 3**.

**Table 3:** Mean differences in infant birth weight in grams per unit increase in average concentrations of maternal vitamin A, D and E in second and third trimester.

N = 57	Mean differences	95% CI	P-value
Mean vitamin A, p-retinol, per 0.1 µmol/L			
Crude	-4.8	-45.0 – 35.4	0.811
Model 1	-11.5	-55.1 – 32.1	0.599
Model 2	-6.0	-45.1 – 33.1	0.759
Mean vitamin D, s-25(OH)D), per 10 nmol/L			
Crude	-35.1	-84.1 – 13.8	0.156
Model 1	-32.7	-81.5 – 16.0	0.184
Model 2	-39.3	-82.5 – 3.9	0.074
Mean vitamin E, p-α-tocopherol, per 10 µmol/L			
Crude	43.5	-109.1 – 196.0	0.571
Model 1	61.6	-99.2 – 222.4	0.445
Model 2	103.0	-46.1 – 252.0	0.171

Mean difference = unstandardized linear regression coefficient β, 95% CI, confidence interval.

p, plasma; s, serum.

Model 1 adjusted for: age (years), pre-pregnancy body mass index (kg/m²), parity (≥1 vs. 0), education (no university vs. university), group allocation from the original RCT (control vs. exercise), and smoking status (yes vs. no). Model 2: Model 1 + gestational weight gain category (above vs. recommended/lower according to Institute of Medicine (IOM) (25), gestational length (weeks), gestational diabetes mellitus (yes in second and/or third trimester vs. neither), and gestational hypertension (yes in second and/or third trimester vs. neither).

**Table 4:** Mean differences in infant birth weight in grams per unit increase in the product of z-scores of mean vitamin A and D concentrations in the second and third trimesters.

N = 57	Mean differences	95% CI	P-value
Mean vitamin A (z-score) x Mean vitamin D (z-score)			
Crude	-35.6	-147.8 – 76.7	0.528
Model 1	-15.5	-129.8 – 98.8	0.786
Model 2	-6.2	-107.1 – 94.7	0.902

Mean difference = unstandardized linear regression coefficient β, 95% CI, confidence interval.

p, plasma; s, serum.

Model 1 adjusted for: age (years), pre-pregnancy body mass index (kg/m²), parity (≥ 1 vs. 0), education (no university vs. university), group allocation from the original RCT (control vs. exercise), and smoking status (yes vs. no). Model 2: Model 1 + gestational weight gain category (above vs. recommended/lower according to Institute of Medicine (IOM) (25), gestational length (weeks), gestational diabetes mellitus (yes in second and/or third trimester vs. neither), and gestational hypertension (yes in second and/or third trimester vs. neither).

The average vitamin A, D, or E concentrations across the second and third trimester were not associated with birth weight in either crude or adjusted regression analyses (**Table 3**). To explore potential synergistic effects between vitamins A and D, we examined their interaction using the product (z-scored) of average vitamin A and D concentrations. This analysis did not reveal any significant associations with birth weight (**Table 4**).

Apart from a weak inverse association between third trimester serum 25(OH)D and birth weight in the crude model, no significant associations between trimester-specific maternal A, D, or E concentrations and birth weight were observed in either crude or adjusted models (**Supplementary Table 3**).

## Discussion

In this study of individuals with pre-pregnancy overweight or obesity, circulating vitamin A and D concentrations declined from mid- to late pregnancy, while vitamin E concentrations increased markedly. Vitamin D insufficiency or deficiency was common. Nearly half of the newborns were macrosomic, reflecting the cohort’s high metabolic risk profile. Despite marked changes in vitamin concentrations during pregnancy and relatively high prevalence of vitamin A and D insufficiency, we found no consistent associations between maternal fat-soluble vitamin concentrations and infant birth weight, either when assessed at individual trimesters, as an average exposure across pregnancy, or when exploring potential synergistic effects of vitamins A and D.

We observed distinct trimester-specific changes in circulating fat-soluble vitamins. Plasma retinol and serum 25(OH)D declined from the second to the third trimester, whereas plasma α-tocopherol increased substantially. The observed changes are in line with several previous studies (6, 33–36). A decrease in vitamin A and D can reflect inadequate nutritional status, but also physiological adaptations of pregnancy, including increased hemodilution and fetal transfer and changes in distribution and utilization (33, 37). The increase in vitamin E throughout pregnancy is due to the increase in lipid concentration, lipoprotein transport and bioavailability during gestation (38–40).

Vitamin A deficiency was not detected in this study. However, 19.3% of the individuals had retinol concentrations classified as insufficient in late pregnancy. This prevalence was substantially lower than in our previous study of a largely normal-weight pregnant population, in which approximately 45% exhibited vitamin A insufficiency or deficiency during the third trimester (unpublished data). In contrast, vitamin D deficiency and insufficiency were common, especially in the third trimester, affecting 31.6% and 33.3% of participants, respectively. This aligns with previous studies and emphasizes that hypovitaminosis D is a widespread condition during pregnancy (41). Overweight and obesity have been consistently linked to vitamin D deficiency (17). However, in the current study, the proportion of participants with serum 25(OH)D < 50 nmol/L in the third trimester was lower than in our previous study of largely normal-weight participants during pregnancy, in which 41% were classified as vitamin D deficient (36).

More than half of the participants exhibited α-tocopherol concentrations above the applied reference range in the third trimester. Interpretation of these values should be approached with caution. Circulating α-tocopherol is transported predominantly in lipoproteins, and pregnancy is characterized by progressive physiological hyperlipidemia, particularly in late gestation (38, 39) Given the lack of pregnancy-specific cut-offs and the strong dependence of circulating α-tocopherol on lipid levels, these elevations likely reflect increases in lipoproteins rather than excessive intake or toxicity. This consideration may be particularly relevant in women with overweight or obesity, in whom altered lipid metabolism may further influence circulating fat-soluble vitamin concentrations (14). In the absence of pregnancy-specific reference ranges and lipid-adjusted cut offs, absolute concentrations may overestimate vitamin E status during late pregnancy. These findings highlight the need for pregnancy-adapted reference values and careful interpretation of fat-soluble vitamin biomarkers during gestation. Notably, vitamin E deficiency during pregnancy is uncommon, and the WHO does not recommend vitamin E supplementation, as current evidence indicates limited or no benefit in reducing adverse maternal or fetal outcomes (42).

Contrary to our hypothesis, maternal circulating levels of vitamins A, D, and E were not associated with birth weight or risk of macrosomia. These findings persisted after adjustment for relevant confounders and mediators. We also found no evidence for a synergistic effect of vitamins A and D on fetal growth. These findings are consistent with previous studies showing no, weak, or inconsistent relationships between maternal fat-soluble vitamins and birth weight (9, 43, 44), but contrast with others reporting significant associations (4, 6, 8–13, 33).

The relationship between fat-soluble vitamins and fetal growth can be threshold-dependent rather than linear. Although insufficiency of vitamins A and D was common in this cohort, no cases of severe deficiency were observed. Birth weight may be affected only when maternal vitamin A or D levels fall into the severely deficient range during pregnancy. Low maternal vitamin A and D status is typically associated with an increased risk of low birth weight, an infant category that was not represented in this cohort. In pregnancies complicated by overweight or obesity, metabolic factors such as insulin resistance, gestational weight gain, and altered placental nutrient transport may play a substantial role in fetal growth and could reduce the relative contribution of micronutrient status. Indeed, nearly three-quarters of the participants in this study exceeded a recommended weight gain during pregnancy, and the prevalence of macrosomia was high (43.9%), supporting the notion that excess energy availability and altered glucose metabolism may dominate fetal growth regulation in this population.

The prevalence of high birth weight and macrosomia in this cohort was elevated compared with general obstetric populations. However, it aligns with findings from high-income countries showing similar rates among individuals with pre-pregnancy overweight or obesity, particularly when excessive gestational weight gain is present (45, 46). The absence of infants with low birth weight further indicates that our cohort represents a metabolically high-risk group rather than a population-based sample. Whereas this limits generalizability to normal-weight pregnancies, it strengthens the clinical relevance of the findings for populations at increased risk of excessive fetal growth and later cardiometabolic disease.

### Strengths and limitations

Strengths of this study include repeated measurements of multiple circulating fat-soluble vitamins across pregnancy in individuals with pre-pregnancy overweight or obesity, the use of gold-standard analytical methods (HPLC and LC-MS/MS), and a detailed characterization of maternal metabolic and pregnancy-related factors. Longitudinal assessment enabled us to capture trimester-specific changes and average exposure, which may better reflect fetal vitamin availability than single time-point measurements typically used in similar studies. We also included an interaction analysis of vitamins A and D.

We acknowledge several limitations to our study. Because this study is a secondary analysis of a cohort that was not originally designed to evaluate the primary exposure-outcome relationship of interest, no *a priori* sample size estimation or power calculation specific to this research question was performed. The relatively modest sample size may therefore have limited ability to detect small but potentially clinically relevant associations. Further, the observational nature of the study precludes causal inference, and residual confounding cannot be excluded. Information on socioeconomic variables was limited, and only education level was included as a confounder. The registration of infant sex was not systematically recorded, and infant sex was therefore not included in the analyses. We did not include dietary and supplement intake in the analysis, which precludes assessment of intake-status relationships. However, circulating concentrations may be more relevant for fetal exposure than intake alone, particularly in obesity, where sequestration and altered metabolism influence bioavailability. Further, while circulating retinol is the most commonly used biomarker of vitamin A in clinical practice, it may not reflect marginal vitamin A status due to homeostatic regulation and limited correlation with intake or clinical symptoms (47, 48). Unfortunately, we did not have access to alternative biomarker measurements (such as circulating retinol-binding protein or isotope-based assessments) nor additional sample material, which limited our ability to more accurately characterize the vitamin A status. Finally, the lack of established pregnancy-specific cut-offs for vitamin E complicates the interpretation of elevated α-tocopherol concentrations in late pregnancy.

## Conclusion

In this cohort of pregnant individuals with pre-pregnancy overweight or obesity, circulating vitamins A and D declined across gestation, whereas vitamin E concentrations increased. A high prevalence of vitamin A insufficiency, vitamin D deficiency and insufficiency, and macrosomia was observed. However, none of the measured fat-soluble vitamins were independently associated with birth weight. These findings indicate that fat-soluble vitamin status in mid- to late pregnancy may not play a major role in determining fetal growth in this metabolically high-risk population. Future studies are warranted to better characterize micronutrient during pregnancy and to establish pregnancy-specific reference ranges, particularly for vitamin E.

## Data Availability

The original contributions presented in the study are included in the article/**Supplementary Material**. Further inquiries can be directed to the corresponding author.
