# Supplementary material for: Maternal fat-soluble vitamin trajectories and infant birth weight in individuals with overweight or obesity
Source: Front Endocrinol (Lausanne). 2026 Apr 15;17:1809102. doi: 10.3389/fendo.2026.1809102 (PMC13124481; doi:10.3389/fendo.2026.1809102)
Supplement: Supplementary file 1 [file DataSheet1.pdf]

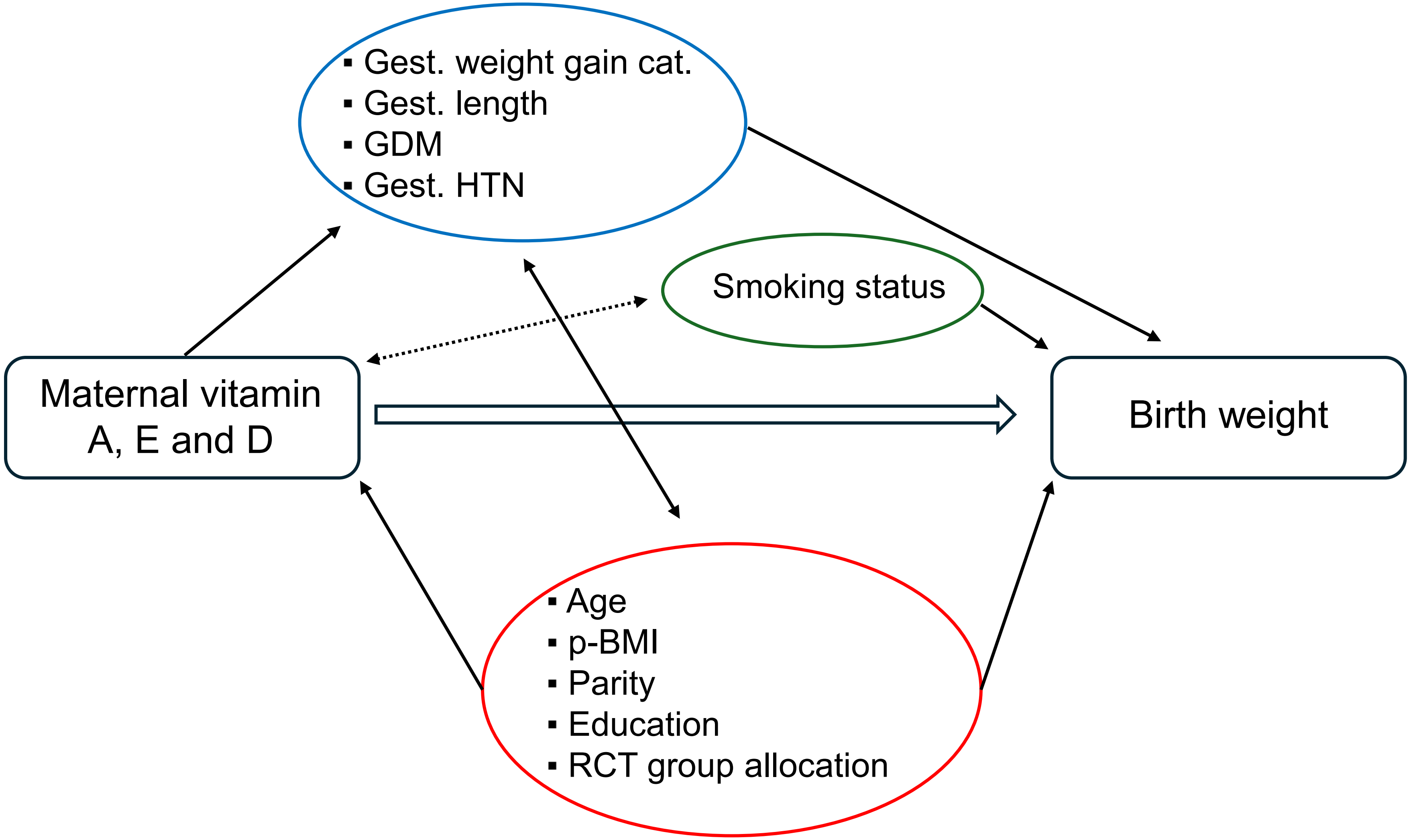

Supplementary Figure 1. Directed acyclic graph (DAG) model for birth weight. Exposure: maternal circulating concentrations of vitamin A, D and E. Confounders (red): maternal age at inclusion in years, pre-pregnancy body mass index (p-BMI) in kg/m^2, parity, education, and group allocation from the original Randomized Control Trial (RCT). Co-variate (green): smoking status. Mediators (blue): gestational weight gain category according to Institute of Medicine (IOM), gestational length, gestational diabetes mellitus (GDM) and gestational hypertension (gest. HTN).
