# Supplementary material for: Maternal fat-soluble vitamin trajectories and infant birth weight in individuals with overweight or obesity
Source: Front Endocrinol (Lausanne). 2026 Apr 15;17:1809102. doi: 10.3389/fendo.2026.1809102 (PMC13124481; doi:10.3389/fendo.2026.1809102)
Supplement: Supplementary file 2 [file Table1.docx]

**Supplementary Table 1. Recommended gestational weight gain guidelines**

|  | **Total weight gain during**  **pregnancy** | **Weight gain during second and third trimesters** |
| --- | --- | --- |
| **Pre-pregnancy body mass index (BMI) categories** | **Range in kg** | **Mean (range) in kg/week** |
| Underweight (< 18.5 kg/m^2^) | 12.5 – 18.0 | 0.51 (0.44 – 0.58) |
| Normal weight (> 18.5 – < 25.0 kg/m^2^) | 11.5 – 16.0 | 0.42 (0.35 – 0.50) |
| Overweight (≥ 25.0 – < 30.0 kg/m^2^) | 7.0 – 11.5 | 0.28 (0.23 – 0.33) |
| Obesity (≥ 30 kg/m^2^) | 5.0 – 9.0 | 0.22 (0.17 – 0.27) |

Recreated from Institute of Medicine (IOM) 2009 gestational weight gain (GWG) Report [Institute of Medicine (US) and National Research Council (US) *Weight Gain during Pregnancy: Reexamining the Guidelines*. Rasmussen KM, Yaktine, AL, editors. Washington DC: The National Academies Press (2009)].
