# Supplementary material for: Maternal fat-soluble vitamin trajectories and infant birth weight in individuals with overweight or obesity
Source: Front Endocrinol (Lausanne). 2026 Apr 15;17:1809102. doi: 10.3389/fendo.2026.1809102 (PMC13124481; doi:10.3389/fendo.2026.1809102)
Supplement: Supplementary file 3 [file Table2.docx]

**Supplementary Table 2. Characteristics stratified for birth weight categories**

|  | **Total**  **N = 57** | **Normal weight**  **N = 32** | **Macrosomia**  **N = 25** |
| --- | --- | --- | --- |
| **Second trimester** |  |  |  |
| p-retinol, µmol/L | 1.55 ± 0.37 | 1.46 ± 0.35 | 1.59 ± 0.39 |
| s-25(OH)D, nmol/L | 63.64 ± 24.96 | 67.76 ± 28.27 | 58.37 ± 19.25 |
| p-α-tocopherol, µmol/L | 34.61 ± 7.14 | 34.00 ± 7.32 | 35.44 ± 6.97 |
| **Third trimester** |  |  |  |
| p-retinol, µmol/L | 1.32 ± 0.33 | 1.34 ± 0.29 | 1.29 ± 0.39 |
| s-25(OH)D, nmol/L | 73.67 ± 30.00 | 71.40 ± 32.02 | 76.59 ± 27.58 |
| p-α-tocopherol, µmol/L | 46.28 ± 10.02 | 45.84 ± 11.35 | 46.84 ± 8.21 |
| **Mean p-retinol, µmol/L** | 1.41 ± 0.29 | 1.40 ± 0.25 | 1.44 ± 0.34 |
| **Mean s-25(OH)D, nmol/L** | 68.66 ± 23.52 | 69.58 ± 26.18 | 67.48 ± 20.07 |
| **Mean p-α-tocopherol, µmol/L** | 40.45 ± 7.67 | 39.91 ± 8.50 | 41.14 ± 6.56 |
| **Maternal age, years** | 31.35 ± 3.47 | 31.59 ± 3.27 | 31.28 ± 3.77 |
| **Pre-pregnancy BMI, kg/m^2,a^** | 34.00 ± 4.60 | 32.98 ± 4.50 | 35.41 ± 4.43 |
| **Gest. weight gain / week, kg*** | 11.77 ± 9.80 | 11.91 ± 6.21 | 11.57 ± 8.60 |
| **Gest. weight gain category**** |  |  |  |
| Under | 6 [10.5] | 3 [9.4] | 3 [12.0] |
| Over | 43 [75.5] | 25 [78.1] | 18 [72.0] |
| Within | 8 [14.0] | 4 [12.5] | 4 [16.0] |
| **Original RCT group** |  |  |  |
| Exercise | 31 [54.4] | 20 [62.5] | 11 [44.0] |
| Control | 26 [45.6] | 12 [37.5] | 14 [56.0} |
| **Maternal age** | 31.6 ± 3.47 | 31.59 ± 3.77 | 31.28 ± 3.77 |
| **Parity ≥ 1** | 30 [52.6] | 13 [40.6] | 17 [68.0] |
| **Smoking^b^** | 8 [14.3] | 6 [19.4] | 2 [8.0] |
| **Gest. length, weeks** | 40.0 (2] | 40 (2) | 40 (1) |
| **GDM***** | 13 [22.8] | 6 [18.8] | 7 [28.0] |
| **G-HTN****** | 11 [19.3] | 6 [18.8] | 5 [20.0] |

Data are in mean ± standard deviation, number [percentage] or median (interquartile range)

Normal weight range: ≥ 2500 – < 4000 g, Macrosomia: ≥ 4000 g

s = serum, p = plasma, BMI = body mass index,

**^a^**Missing data from N = 2, **^b^**Missing data from N = 1

*Weight gain per week in kg from second trimester to pre-delivery

**Gestational weight gain category based on pre-pregnancy body mass index (BMI) as recommended by Institute of Medicine (IOM) guidelines [Institute of Medicine (US) and National Research Council (US) *Weight Gain during Pregnancy: Reexamining the Guidelines*. Rasmussen KM, Yaktine, AL, editors. Washington DC: The National Academies Press (2009)].

***GDM = Gestational Diabetes Mellitus: fasting plasma glucose ≥ 7.0 mmol/L, and/or 2 hours glucose ≥ 11.1 mmol/L after an Oral Glucose Tolerance Test

****HTN = Gestational Hypertension: systolic blood pressure ≥ 140 mmHg and/or diastolic blood pressure ≥ 90 mmHg
