# Supplementary material for: Maternal fat-soluble vitamin trajectories and infant birth weight in individuals with overweight or obesity
Source: Front Endocrinol (Lausanne). 2026 Apr 15;17:1809102. doi: 10.3389/fendo.2026.1809102 (PMC13124481; doi:10.3389/fendo.2026.1809102)
Supplement: Supplementary file 4 [file Table3.docx]

**Supplementary Table 3. Mean differences in birth weight in grams per unit increase of maternal vitamin A, D and E concentrations during second and third trimesters**

| **N = 57** | **Mean difference** | **95% CI** | **p-value** |
| --- | --- | --- | --- |
| **Vitamin A, p-retinol,**  **per 0.1 µmol/L** |  |  |  |
| **Second trimester** | 15.0 | -16.2 – 46.2 | 0.339 |
| Model 1 | 10.3 | -23.6 – 44.2 | 0.543 |
| Model 2 | 9.1 | -25.7 – 43.9 | 0.602 |
| **Third trimester** | -26.2 | -60.6 – 8.3 | 0.134 |
| Model 1 | -25.4 | -61.6 – 10.7 | 0.164 |
| Model 2 | -26.8 | -63.5 – 9.9 | 0.148 |
| **Vitamin D, s-25(OH) D,**  **per 10 nmol/L** |  |  |  |
| **Second trimester** | -11.5 | -50.1 – 27.4 | 0.555 |
| Model 1 | -10.3 | -48.9 – 28.3 | 0.594 |
| Model 2 | -9.9 | -49.4­ – 29.6 | 0.617 |
| **Third trimester** | -45.7 | -91.0 – 0.3 | 0.048 |
| Model 1 | -39.9 | -85.5 – 5.7 | 0.085 |
| Model 2 | -38.5 | -84.9 – 7.9 | 0.102 |
| **Vitamin E, p-α-tocopherol, per 10 µmol/L** |  |  |  |
| **Second trimester** | 70.0 | -93.2 – 233.2 | 0.393 |
| Model 1 | 84.8 | -93.8 – 263.5 | 0.345 |
| Model 2 | 82.2 | -104.1 – 268.5 | 0.379 |
| **Third trimester** | 15.3 | -101.7 – 132.3 | 0.794 |
| Model 1 | 31.2 | -87.8 – 150.2 | 0.601 |
| Model 2 | 15.8 | -109.5 – 141.1 | 0.801 |

Mean difference = unstandardized linear regression coefficient β, CI = confidence interval. p = plasma, s = serum. Model 1 adjusted for: maternal age at inclusion (years), pre-pregnancy body mass index (kg/m²), parity (≥ 1 vs. 0), group allocation from the original RCT (control vs. exercise), and smoking status (yes vs. no). Model 2: Model 1 + gestational weight gain category (above vs. recommended/lower according to Institute of Medicine (IOM) guidelines [Institute of Medicine (US) and National Research Council (US) *Weight Gain during Pregnancy: Reexamining the Guidelines*. Rasmussen KM, Yaktine, AL, editors. Washington DC: The National Academies Press (2009)], gestational length (weeks), gestational diabetes mellitus (yes in second and/or third trimester vs. neither), and gestational hypertension (yes in second and/or third trimester vs. neither).
